# Supplementary figures and images for: Reducing alcohol consumption in UK armed forces veterans: Feasibility of using personalized push notifications with AI
Source: PLOS Digit Health. 2026 Apr 10;5(4):e0001322. doi: 10.1371/journal.pdig.0001322 (PMC13068231; doi:10.1371/journal.pdig.0001322)

**S1 Fig: Training results**


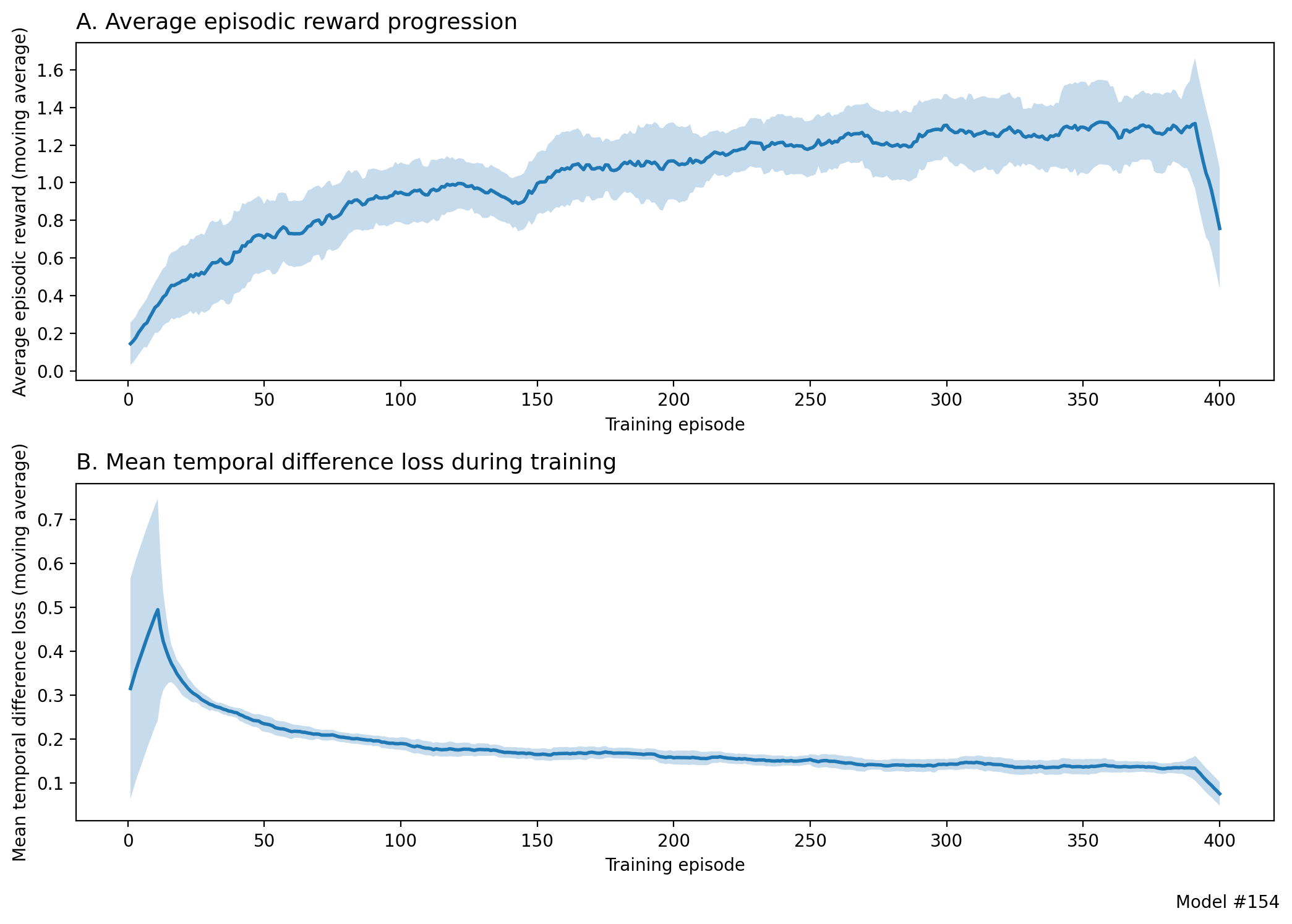

Supplement: S1 Fig — (DOCX) [file pdig.0001322.s001.docx]
