# Supplementary material for: Reducing alcohol consumption in UK armed forces veterans: Feasibility of using personalized push notifications with AI
Source: PLOS Digit Health. 2026 Apr 10;5(4):e0001322. doi: 10.1371/journal.pdig.0001322 (PMC13068231; doi:10.1371/journal.pdig.0001322)
Supplement: S1 Table — (DOCX) [file pdig.0001322.s002.docx]

**S1 Table: Baseline characteristics of ≥6 weeks vs <6 weeks.**

| **Variable** | **Overall (*n*=2120)** | **≥6 weeks**  **(*n*=728)** | **<6 weeks**  **(*n*=1392)** |
| --- | --- | --- | --- |
| Age in years (Mean, SD) | 50.2 (19.79) | 48.4 (19.26) | 51.2 (20.01) |
| Gender (n, %)  Male  Female | 1850 (87.3)  270 (12.7) | 97 (13.3)  631 (86.7) | 1219 (87.6)  173 (12.4) |
| Service branch (n, %)  Army  Royal Air Force  Royal Navy | 1606 (75.8)  269 (12.7)  245 (11.6) | 556 (76.4)  82 (11.3)  90 (12.4) | 1050 (75.4)  187 (13.4)  155 (11.1) |
| Phone OS (n, %)  iOS  Android | 1315 (62.0)  805 (38.0) | 433 (59.5)  295 (40.5) | 882 (63.4)  510 (36.6) |
| Prior alcohol treatment (n, %)  No  Yes | 1869 (88.2)  251 (11.8) | 636 (87.4)  92 (12.6) | 1233 (88.6)  159 (11.4) |
