# Supplementary material for: Reducing alcohol consumption in UK armed forces veterans: Feasibility of using personalized push notifications with AI
Source: PLOS Digit Health. 2026 Apr 10;5(4):e0001322. doi: 10.1371/journal.pdig.0001322 (PMC13068231; doi:10.1371/journal.pdig.0001322)
Supplement: S2 Table — (DOCX) [file pdig.0001322.s003.docx]

**S2 Table: Effect size estimates.**

Effect size estimates for planning: weekly alcohol units (personalized minus no personalization)

| **Week** | **No personalisation, estimate (95% CI)** | **Personalised, estimate (95% CI)** | **Difference (personalised − no), estimate (95% CI)** |
| --- | --- | --- | --- |
| 0 | 31.25 (29.69, 32.80) | 31.08 (29.44, 32.73) | -0.16 (-2.43, 2.10) |
| 1 | 27.82 (26.27, 29.37) | 25.26 (23.61, 26.91) | -2.56 (-4.82, -0.30) |
| 2 | 25.63 (24.08, 27.19) | 20.88 (19.23, 22.52) | -4.76 (-7.02, -2.49) |
| 3 | 20.92 (19.36, 22.47) | 17.83 (16.19, 19.48) | -3.08 (-5.35, -0.82) |
| 4 | 18.21 (16.65, 19.77) | 16.14 (14.49, 17.79) | -2.07 (-4.34, 0.20) |
| 5 | 15.70 (14.14, 17.26) | 13.92 (12.25, 15.58) | -1.78 (-4.06, 0.50) |
| 6 | 17.25 (15.34, 19.16) | 16.76 (14.78, 18.74) | -0.49 (-3.24, 2.27) |
| 7 | 18.45 (16.21, 20.69) | 16.47 (14.30, 18.65) | -1.98 (-5.10, 1.14) |
| 8 | 16.69 (14.07, 19.32) | 15.07 (12.61, 17.53) | -1.62 (-5.22, 1.97) |
| 9 | 18.21 (15.23, 21.20) | 14.22 (11.60, 16.85) | -3.99 (-7.97, -0.02) |
| 10 | 17.26 (13.88, 20.63) | 14.13 (11.16, 17.11) | -3.12 (-7.62, 1.37) |
| 11 | 15.18 (11.78, 18.58) | 13.21 (10.25, 16.17) | -1.97 (-6.47, 2.54) |

Effect size estimates for planning: weekly app usage (seconds; personalized minus no personalization).

| **Week** | **No personalisation, estimate (95% CI)** | **Personalised, estimate (95% CI)** | **Difference (personalised − no), estimate (95% CI)** |
| --- | --- | --- | --- |
| 0 | 177.92 (173.14, 182.70) | 207.04 (201.98, 212.10) | 29.12 (22.15, 36.08) |
| 1 | 183.68 (178.90, 188.46) | 212.39 (207.32, 217.45) | 28.71 (21.75, 35.67) |
| 2 | 178.14 (173.36, 182.92) | 192.09 (187.03, 197.15) | 13.95 (6.99, 20.91) |
| 3 | 179.21 (174.43, 183.99) | 203.12 (198.05, 208.19) | 23.91 (16.94, 30.88) |
| 4 | 169.17 (164.35, 173.98) | 176.22 (171.15, 181.29) | 7.06 (0.07, 14.05) |
| 5 | 171.68 (166.87, 176.49) | 194.27 (189.15, 199.40) | 22.60 (15.57, 29.62) |
| 6 | 165.59 (159.59, 171.58) | 171.00 (164.81, 177.20) | 5.42 (-3.20, 14.04) |
| 7 | 155.92 (148.82, 163.02) | 144.06 (137.21, 150.91) | -11.86 (-21.73, -1.99) |
| 8 | 150.47 (142.09, 158.85) | 159.27 (151.46, 167.07) | 8.80 (-2.65, 20.25) |
| 9 | 130.98 (121.40, 140.55) | 128.93 (120.58, 137.29) | -2.05 (-14.75, 10.66) |
| 10 | 101.04 (90.18, 111.90) | 117.15 (107.62, 126.68) | 16.11 (1.66, 30.56) |
| 11 | 103.90 (92.96, 114.83) | 116.43 (106.95, 125.90) | 12.53 (-1.94, 27.00) |
