# Supplementary material for: Reducing alcohol consumption in UK armed forces veterans: Feasibility of using personalized push notifications with AI
Source: PLOS Digit Health. 2026 Apr 10;5(4):e0001322. doi: 10.1371/journal.pdig.0001322 (PMC13068231; doi:10.1371/journal.pdig.0001322)
Supplement: Attachment — Submitted filename: response-reviewers.docx [file pdig.0001322.s005.docx]

January 23, 2026

Dear Colleagues,

**RE: Submission of consideration - Response to Reviewers**

Please find enclosed our responses to the comments raised by the Reviewers. We are grateful for the time they have taken to review our manuscript. We have provided a tracked-changed version, and a clean version of our manuscript. Page numbers refer to the clean/tracked version of the manuscript.

We have amended the title to reflect the work present within the manuscript; and considering reviewer feedback. The title has been changed to: “Reducing alcohol consumption in UK Armed Forces veterans: feasibility of using personalized push notifications using AI”.

We have also amended the stated Conflicts of Interest and Contributions sections. These changes have been reported in the Editorial Management system and cover letter. In addition, we have made typographical improvements throughout the manuscript.

**General comments**

Comment: Data availability expectations (PLOS Data policy): ensure all data underlying findings are fully available without restriction, or clearly specify any restrictions (e.g. privacy, third-party data) and how access can be requested.

Response: The aggregated dataset used in this analysis will be available via the Open Science Framework. Please see Page 2, Paragraph 2.

**Reviewer 1**

Comment (reviewer 1): Table 5 shows the no personalisation group received far fewer notifications than the personalisation group, creating a confound (personalisation effect vs message dose).

Response: The difference in the total number of notifications between groups is expected and reflects the intervention definition rather than an unintended imbalance. Both groups received the same generic notifications (scheduled prompts and reminders), whereas the personalisation group additionally received personalised and event-triggered messages selected by the framework in response to each participant’s adaptive persona and recent behaviour.

As Table 5 highlights, the groups received a similar number of generic notifications, and the higher total in the personalisation group is attributable to the added personalised and personalised (event) notifications that are important to the intervention being evaluated.

We have clarified this explicitly in the Methods (page 7) and Results (page 14) and strengthened the Discussion (page 21) to state that this feasibility evaluation compares standard generic messaging with an enhanced AI-supported messaging package (content plus timing and delivery strategy). As a result, the study is not intended to isolate the independent contribution of message dose versus personalisation content, and a future factorial design (or similar approach) would be required to disentangle these components.

Comment (reviewer 1): Provide more detail on training and deployment of the reinforcement learning model: training set size, synthetic data, researcher-curated data, training metrics (e.g. loss), which reinforcement learning algorithm, whether reinforcement learning is necessary, and whether the model adapted during deployment or was trained offline.

Response: We have added a short implementation detail to the Methods for reproducibility. The reinforcement learning component is a deep q network (DQR) agent that selects the next message category as a sequential decision problem, using the feature set outlined in Table 1 and the adaptive persona as state inputs.

Training data comprised three sources already described in the manuscript; historical app interaction data, synthetic episodes generated to broaden behavioural coverage where real-world interactions were very sparse, and researcher-curated message-interaction scenarios. Model selection used *k*-fold cross-validation with hyperparameter optimisation and early stopping when reward accumulation plateaued; we additionally report training diagnostics (average episodic reward progression and mean temporal difference loss) in Supplementary 1.

Regarding deployment, we clarify that the DQN was trained offline and deployed as a fixed policy within a cloud-based infrastructure. The model parameters were not updated during participant follow-up; adaptation occurs via the rolling 28-day adaptive persona and the exploration/exploitation strategy, with rule-based guardrails and fallback to generic messaging when insufficient data are available.

**Reviewer 2**

Comment (reviewer 2): Lack of description of AI personalisation algorithm performance metrics (e.g. accuracy, reward progression).

Response: The manuscript already describes the key reinforcement learning performance metric used for this model: we tracked average episodic reward during training and applied early stopping when reward accumulation plateaued. Because this is a DQN reinforcement learning formulation, “accuracy” is not an appropriate metric (the objective is to maximise expected reward rather than predict labelled outcomes). To make this clearer, we have added a sentence in the Methods signposting the training diagnostics and provided the reward progression (and temporal difference loss) in the Supplementary 1.

Comment (reviewer 2): Unclear why there is no comparison between participants who dropped out and those who completed at least 6 weeks of app usage.

Response: We agree that this comparison is useful to assess potential selection effects arising from the ≥6-week usage threshold. We have therefore added Supplementary 2 presenting baseline characteristics for participants with ≥6 weeks of app usage (n=728) versus those with <6 weeks (n=1,392). Baseline characteristics by ≥6-week eligibility are provided in Supplementary 2.

Comment (reviewer 2): Unclear why mental health comorbidities and socioeconomic factors were not included or adjusted beyond age, gender, and prior treatment.

Response: This feasibility evaluation used a minimal, pre-specified adjustment set (age, gender, and prior alcohol treatment) to support model stability and interpretability rather than to make strong causal claims. Socioeconomic indicators were not available in the dataset and therefore could not be included. Mental health symptom/comorbidity measures were not included in the primary outcome models because these data were not collected consistently across users and had substantial missingness in the data used for model development and evaluation; additionally, mental health symptoms may plausibly lie on the causal pathway, such that adjustment could risk over-adjustment. We have clarified this rationale in the manuscript.

Comment (reviewer 2): Include subgroup analyses (e.g. gender, age, prior treatment) to explore differential effects.

Response: We agree that differential effects by gender, age, and prior treatment are important research questions. However, this study was designed as a feasibility evaluation and was not powered to support reliable subgroup or interaction analyses; conducting multiple subgroup comparisons would risk spurious findings and over-interpretation. We have therefore not added subgroup analyses in this manuscript and instead note subgroup effects as a priority for a future adequately powered trial.

Comment (reviewer 2): Significant differences at some weeks but overall trend converges by Week 12; this needs further discussion.

Response: We agree and have expanded the Discussion to interpret the temporal pattern. The personalised group showed earlier reductions and several week-specific between-group differences, but trajectories converged by Week 12. This pattern is consistent with an early intervention response followed by attenuation over time, which may reflect diminishing marginal effects of repeated messaging, habituation to notifications, changes in engagement across follow-up, and/or regression towards a stable level of consumption in both groups.

Comment (reviewer 2): Report effect sizes to support planning future large clinical trials and sample size estimation.

Response: We agree. To support planning of a future fully powered trial and sample size estimation, we now report week-specific between-group effect size estimates (personalised minus no personalisation) with 95% CIs for both outcomes. These are provided as Supplementary 3. As this is a feasibility evaluation, these effect sizes are presented for planning purposes rather than definitive inference.

Comment (reviewer 2): Are there data on user satisfaction or perceived relevance of messages? Qualitative feedback on use or non-use would be helpful.

Response: We collected user satisfaction and perceived relevance data, including in-app message ratings (and optional qualitative feedback). These data are being analysed and will be reported in a separate, dedicated paper focused on acceptability and user experience to allow appropriate depth and interpretation. This feasibility manuscript therefore concentrates on usage and behavioural outcomes rather than acceptability outcomes. This is to ensure it remains concise.

Comment (administrative decision point): PLOS offers the option to publish the peer review history; authors must decide whether to opt in.

Response: We agree to opt in to this process.

We look forward to receiving your decision.

Best wishes

Dr Daniel Leightley

On behalf of all authors.
